# RNA Splicing in Cancer and Targeted Therapies

**DOI:** 10.3390/genes14112020

**Published:** 2023-10-29

**Authors:** Md Rafikul Islam, Preeti Nagar, Shegufta Tasneem Neetole, Ledong Wan, Mohammad Alinoor Rahman

**Affiliations:** 1 Department of Biochemistry and Molecular Biology, University of Arkansas for Medical Sciences, Little Rock, AR 72205, USA; mrislam@uams.edu (M.R.I.); pnagar@uams.edu (P.N.); stneetole@uams.edu (S.T.N.); 2 Winthrop P. Rockefeller Cancer Institute, University of Arkansas for Medical Sciences, Little Rock, AR 72205, USA; 3 Cold Spring Harbor Laboratory, Cold Spring Harbor, NY 11724, USA

Since the discovery of RNA splicing as a fundamental step to remove introns from pre-mRNA to produce mature mRNAs, substantial research in the past decades has highlighted RNA splicing as a critical mediator of gene expression and proteome diversity, also being important in many developmental and biological processes. In addition, critical insights have transpired regarding the interplay between splicing and other steps in mRNA metabolism, including transcription and the various relevant features of chromatin biology, processing at the 5′ and 3′ ends, RNA editing or modifications, mRNA export and localization, and mRNA turnover or decay. Genetic and cellular alterations impairing the correct complements of RNA and RNA-binding proteins compromise the fidelity of splicing and splicing-associated processes and often cause pathological consequences, including genetic defects, neurological disorders, and cancer [1,2]. Advances in high-throughput sequencing have revealed that dysregulated RNA splicing affects almost every hallmark of tumorigenesis [1,2]. Splicing alteration can induce tumorigenesis via diverse mechanisms, such as augmented cell proliferation, impaired apoptosis, enhanced migration and metastatic potential, resistance to therapy, and the avoidance of immune surveillance. We have compiled this Special Issue of the *Genes* journal focusing on our current understanding of RNA splicing alterations associated with tumor initiation, progression, current and emerging technologies targeting RNA splicing for cancer therapy, critical roadblocks and technical problems, and future challenges in the field.

## 1. Splicing Factor Dysregulation in Cancer

Wan et al., in their review [3], highlighted the roles of serine/arginine-rich (SR) splicing factors in cancer. SR proteins are a family of ultra-conserved and structurally related proteins with critical roles in spliceosome assembly and splicing regulation. In addition, SR proteins function in multiple other RNA-processing-related activities, such as genome stability, RNA export, and translation. SR proteins are frequently upregulated in different tumors, and even slight upregulation often causes them to function as oncoproteins. Upregulated SR proteins cause genome-wide splicing alterations to their target genes, some of which are critically linked to tumorigenesis. In contrast to SR protein over-expression, recurrent mutations are rarely identified in SR proteins, except for SRSF2. Mutations in SRSF2 frequently occur in hematologic malignancies, including myelodysplastic syndromes (MDS) and leukemia, also reported in solid tumors. Mutations in SRSF2 present gain-of-function activity by changing their binding preference for a specific motif in the RNA. Therefore, mutant SRSF2 binds to new target genes and promotes splicing alterations and functional defects in hematopoiesis. Surprisingly, the reasons recurrent mutations are specifically restricted to SRSF2 compared to other SR proteins and why they are frequently observed in hematopoietic tissues have remained elusive. Besides splicing, SR proteins could also exert tumorigenic roles via differential regulation in genome stability, transcription, RNA export, and translation. One interesting avenue is epigenetic regulation. For example, histone methylation alters the affinity of SR proteins to their RNA binding targets, therefore providing a critical link between splicing and epigenetic regulation in tumorigenesis. 

## 2. RNA Editing

RNA editing is an important type of post-transcriptional processing, which changes the primary RNA sequence through the insertion/deletion or modification of specific nucleotides. Piazzi et al., in their review [4], highlighted RNA editing and its link with pathophysiology. The most common modification in humans is deamination. In addition to altering RNA secondary structure, protein binding preferences, or protein-coding sequences, RNA editing may affect RNA splicing by affecting the splice donor or splice acceptor site. Therefore, the enzymes that carry out RNA editing activities have the potential to radically promote transcript diversity, which could be further regulated spatiotemporally, such as in a tissue-specific or disease-specific manner, or in response to cellular stimuli or signaling. Moreover, the enzymes that carry out these modifications are also associated with genomic stability and DNA repair. Another interesting RNA processing step is N6-methyladenosine (m6A) modification, which is also often implicated to affect alternative splicing in cancer. m6A modification is catalyzed by a multi-component enzyme complex including methyltransferase-like 3 (METTL3), methyltransferase-like 14 (METTL14), Wilms tumor suppressor 1-associated protein (WTAP), zinc finger CCCH domain-containing protein 13 (ZC3H13), and methyltransferase-like 16 (METTL16). The upregulation of METTL3 increases m6A levels and significantly affects the progression of hepatocellular carcinoma (HCC). Xu et al., in their article [5], investigated alternative splicing variants of METTL3. They found that a truncated METTL3 variant METTL3-D is less present in HCC tumors than the full-length variant METTL3-A. They further showed that METTL3-D functions as a tumor suppressor, which decreases cellular m6A modification and inhibits proliferation and migration, and the invasion of HCC cells.

## 3. Nonsense-Mediated mRNA Decay (NMD)

NMD is another post-transcriptional RNA processing mechanism. It was initially identified as a surveillance mechanism to safeguard the fidelity of mRNA transcripts. NMD ensures the selective recognition and rapid degradation of erroneous transcripts containing a premature translation-termination codon (PTC). One third of mutated and disease-causing mRNAs were reported to be recognized and degraded by NMD, highlighting the importance of this intricate mechanism in maintaining cellular integrity. In addition to surveillance, NMD also elicits the decay of many endogenous mRNAs (~10% of the human transcriptome) without mutations, therefore also fine-tuning transcript abundance. It is interesting to note that about one third of all alternative splicing (AS) events result in PTC-containing transcripts and are subsequently degraded by NMD. Substantial evidence has disclosed a coordinated regulatory link between AS and NMD to regulate gene expression. This coordinated action is commonly termed as AS coupled to NMD (AS–NMD), which controls the ratio of productive to unproductive transcript isoforms. By modulating gene expression, NMD regulates diverse biological functions during development and differentiation and expedites cellular responses to adaptation, physiological changes, stresses, environmental insults, etc. NMD is a complex pathway regulated by the dynamic interactions between mRNA and a series of NMD-associated proteins and upon a complex choreography of events between mRNA-bound proteins. AS–NMD is further regulated by the splicing pathway. Mutations or alterations (such as abnormal expression, degradation, post-translational modification, etc.) that impair the function or expression of proteins associated with the NMD (or AS–NMD) pathway can be detrimental to cells and may cause pathological consequences. Nagar and Islam et al., in their review [6], extensively highlighted the implications of NMD in developmental and intellectual disabilities, genetic defects, and cancer. Accumulating evidence over the past decades has disclosed NMD as a critical mediator of tumorigenesis. Tumor cells differentially exploit NMD to support their survival, uncontrolled growth, and progression. For example, some tumors degrade a subset of mRNAs via NMD, such as those encoding tumor suppressors, RNA binding proteins, splicing factors, stress response proteins, signaling proteins, and immunogenic neoantigens. In contrast, some tumors inhibit NMD to enhance the expression of oncoproteins or other proteins beneficial for tumor growth and progression. Unraveling how NMD affects tumorigenesis differentially is critical in understanding the complex biological mechanisms of cancer, and it is essential for the development of effective and targeted therapeutic opportunities for personalized medicine.

## 4. Challenges in Identifying Tumorigenic RNA Isoforms 

One critical aspect of targeting RNA splicing for cancer therapy is to identify potential target gene(s) associated with tumorigenesis. Unlike monogenic genetic defects, many genes are simultaneously dysregulated at the splicing level in cancer due to heterogeneous and complex genetic backgrounds. Among these dysregulated splicing events, only a few are directly associated with tumorigenesis (cancer driver events), and the remaining have no relevance to cancer (passenger events). Advancements in high-throughput sequencing, including whole genome sequencing, whole exome sequencing, whole transcriptome sequencing, and non-coding RNA sequencing, and the development of these techniques to identify modified nucleic acids have helped us to identify many cancer-driver splicing events. In addition, they have permitted a global analysis of gene expression, identified novel pathway interactions, and provided critical insights assessing both disease progression and therapeutic response. However, there are significant caveats to these analyses, which are crucial roadblocks against our progress in obtaining the complete picture. Piazzi et al., in their review [4], discussed this issue and highlighted several problems. One of the biggest issues in interpreting RNA-seq data is the absence of many alternatively spliced transcript isoforms in the databanks. This problem appears due to a lack of continuous updates in the databanks, as well as the inability of the research community to upload novel transcripts in a timely manner. The transcriptome-wide detection of alternative splicing isoforms conventionally uses short-read RNA-seq. However, in short-read RNA-seq analyses, it is challenging to accurately reconstruct and quantify entire or full-length isoform expression and understand multiple splicing events in the same gene if they happen among distant exons. Long-read RNA sequencing facilitates reconstructing full-length RNA isoforms, providing critical insights to understand complex alternative splicing, such as multiple splicing events, alternative first or last exon, novel 5′ or 3′ untranslated regions, gene fusions, as well as RNA modification. The major limitation in long-read RNA sequencing is a lower yield (i.e., few reads per sample), which affects its utility for isoform quantification. One option to address this issue is targeted long-read sequencing, which enriches specific isoforms of interest with probe capture or the depletion of high-abundance RNAs. Another option could be combining both long-read and short-read sequencing, where long-read can allow efficient isoform identification and short-read can aid in efficient isoform quantification. 

## 5. Therapeutic Development Targeting RNA Splicing in Cancer

Considering the importance of RNA splicing dysregulation in cancer initiation and progression, there is immense interest in targeting RNA splicing for cancer therapy. Bonner et al., in their review [7], extensively discussed different therapeutic modalities in varying stages of preclinical and clinical development. These include targeting the core spliceosome with small molecule inhibitors, targeting splicing regulatory proteins and/or RNA-binding proteins, modulating specific alternative splicing events, and targeting immunogenic neoantigens. These drugs were employed to inhibit splicing at a global level and, therefore, lacked target specificity with the potential for off-target cytotoxic effects. Interestingly, several studies revealed that cancer cells are more sensitive to these drugs compared to normal cells. Later research developed more efficient synthetic analogues (e.g., E7107), which showed promising results in human tumor xenograft models. Unfortunately, the clinical trial of E7107 was terminated early due to temporary or permanent visual loss in two patients. Due to the inherent off-target risks and cytotoxic effects of global splicing inhibitory drugs, gene-specific targeted therapeutic approaches have received more attention. Oligonucleotide-based pharmacology, e.g., decoy oligonucleotides or antisense oligonucleotides (ASOs), enables the targeting of disease-causing isoforms with a high level of specificity. Oligonucleotides could be chemically modified to enhance binding affinity to the target RNA, cellular delivery, and stability. These modifications subsequently enhance the efficacy of the splicing-modulating activity of oligonucleotides. Several studies reported promising outcomes for oligonucleotides in preclinical models to correct aberrant splicing. Although chemical compound-based splicing inhibitors and oligonucleotide-based approaches offer discrete advantages in targeting aberrant splicing in cancers, their efficacy is significantly affected due to the lack of target specificity and scalability. New technologies are being developed to address such problems. For example, the neomorphic effects of splicing mutations could be leveraged to kill cancer cells containing the mutation selectively. A fascinating modality was recently described [8] for cancer cells with SF3B1 mutation, where six candidate exons were inserted in the herpes simplex virus-thymidine kinase (HSVTK). The treatment of HSV-TK-expressing cells with the antiviral prodrug ganciclovir causes cytotoxic metabolite production, leading to cell death. Gene editing via CRISPR-based approaches is also emerging to modulate specific alternative splicing events. By engineering specific mutations, it is possible to strengthen or abolish a specific splicing regulatory site in a target of interest, thereby promoting exon inclusion or skipping. Alternatively, targeted exon deletions with CRISPR–Cas9 using paired gRNAs can also enable exon skipping from a gene of interest, which could be advantageous for poison exons [1]. 

To conclude, research on RNA splicing should be continued with creativity, novel discoveries, and the courage to challenge existing roadblocks or limitations, which will likely advance the journey of RNA therapeutics in cancer, revolutionizing novel tools for medicine and science.
